# Genetic analysis of the *single internode dwarf 1* mutant in barley

**DOI:** 10.1186/s12870-025-06790-6

**Published:** 2025-07-02

**Authors:** Megan Overlander-Chen, Jason D. Fiedler, Shaobin Zhong, Shengming Yang

**Affiliations:** 1 USDA-ARS Cereals Research Improvement Unit, Edward T. Schafer Agriculture Research Center, Fargo, ND 58102 USA; 2 Department of Plant Sciences, North Dakota State University, Fargo, ND 58102 USA; 3 USDA-ARS Cereal Disease Laboratory, St. Paul, MN 55108 USA; 4 Department of Plant Pathology, North Dakota State University, Fargo, ND 58102 USA

**Keywords:** Sid1, Stem development, Genetic mapping, Barley

## Abstract

**Background:**

Stem development is crucial for plant lodging, nutrients and water transport, and structural support for other organs. Understanding stem development and growth is essential for ensuring global food security. Although numerous lodging-resilient and high-yielding crop varieties have been developed in the Green Revolution by controlling plant height, the molecular mechanism underlying stem development, particularly for cereals, is not fully understood. The allelic stem mutants in barley (*Hordeum vulgare* subsp. *vulgare*), *single internode dwarf 1* (*sid1*), provide a model system for genetic studies on stem development.

**Results:**

We characterized and genetically analyzed the *sid1.b* mutation. To determine the precise position of *Sid1*, a high-resolution genetic map was constructed. Segregating F_2_ plants derived from a cross between wild type (WT) and the mutant were genotyped with the barley 50 k iSelect SNP Array, and the detected SNPs were converted to PCR-based markers for fine mapping. The *Sid1* gene was mapped to a 429-kb region on chromosome 4H. Illumina sequencing of WT and *sid1* identified a C → T transition in an epidermal pattern factor (EPF)-coding gene, which introduces a premature stop codon in the mutant allele.

**Conclusions:**

In the present study, we genetically characterized and mapped the *sid1.a* mutation, which causes a dwarfed phenotype with single internode stems in barley. The EPF-encoding gene in the delimited region is a promising candidate for *Sid1*. Therefore, our study provides a foundation for cloning of *Sid1*, which will enhance our understanding of the molecular mechanisms underlying stem development, particularly in monocot plants.

**Supplementary Information:**

The online version contains supplementary material available at 10.1186/s12870-025-06790-6.

## Background

The plant stem is a crucial organ that provides structural support for leaves, flowers, and fruits while facilitating the transport of water, gases, nutrients, and carbohydrates between the roots and leaves. Stem elongation allows for inflorescence escaping from soilborne pests and microbes, reaching more sunlight, and disseminating seed more distantly [1]. Additionally, stem development determines plant height and shoot architecture, which influence lodging resistance and grain yield. Therefore, understanding stem development and growth is essential for food security for the increasing global population. Although numerous lodging-resilient and high yielding crop varieties have been bred in the Green Revolution through manipulating plant height [2], the molecular mechanism underlying stem development, particularly for cereals, is not fully understood.

Stem development occurs in two distinct stages, primary organogenesis and secondary stem elongation. In comparison with dicots, the grasses with sheathing leaf bases, such as wheat, rice, maize, and barley, rely on a specialized intercalary meristem for stem elongation. In *Arabidopsis*, cell differentiation and active mitotic cell proliferation in the shoot apical meristem (SAM) activates organogenesis and displaces daughter cells downward to extrude the shoot tip [3]. Although the primary stem morphogenesis is also initiated by the SAM activity at the apex, the grasses undergo the secondary stem elongation to lift their inflorescences off the ground primarily through the intercalary cell division [4]. The intercalary meristem locates just above each node, and the daughter cells are moved upward through successive zones of expansion and maturation to form a single internode [5]. Reduced intercalary meristem cell numbers are associated with dwarfism in grasses, highlighting the importance of sufficient founder cells for proper stem elongation [2]. Once intercalary proliferation ceases, further stem growth depends on internode cell elongation.

Although internode elongation is universally regulated by intercalary activity, the timing and signaling mechanisms may differ across internodes. In cereals (dicots also), the internode elongates acropetally from the bottom up until the uppermost internode or peduncle attains the full extension at anthesis [6]. The acropetal pattern may respond to the acropetally distributed and transported Gibberellic acid (GA) along the stem [7, 8]. In comparison to other internodes, the peduncle seems to respond differentially when certain genes regulating stem development are missing. In rice, the *Elongated Uppermost Internode 1* (*EUI1*) gene encodes a cytochrome P450 monooxygenase (CYP450) which deactivates GA specifically in the internode [9, 10]. The recessive *eui1* mutation doubles the length of the peduncle, but causes little or no effect on the other internodes [11]. Similarly, the barley spontaneous mutant *Sheathed spike 1* (*SS1*) exhibits an extremely shortened peduncle resulting a flag leaf sheath-wrapped spike, but other internodes remain unaffected [12].

Even in dwarf mutant caused by abnormal phase transition, differential growth suppression among internodes is evident. The conserved antagonism between microRNA156 (miR156) and miR172 regulates developmental phase transitions in plants by targeting Squamosa *Promoter Binding Like* (*SPL*) and *APETALA2-like* (*AP2-like*) genes, which govern juvenile and reproductive growth, respectively, [13–16]. The barley semi-dwarf *zeocriton 1.b* (*zeo1.b*) results from disrupted miR172-regulation of *HvAP2*. The overall growth of *zeo1.b* internodes is reduced and proceeds more slowly. However, the peduncle was the most stunted, and the elongation of other internodes is less affected [17]. These findings suggest that an additional layer of regulatory signaling may specifically control peduncle elongation. Understanding how plants coordinate these signals to optimize stem architecture is not only a fundamental biological question but also has direct implications for yield improvement.

Barley (*Hordeum vulgare* subsp. *vulgare*) is the fourth most important cereal crop and serves as a valuable monocot model for genomic research. A series of near-isogenic lines (NILs) developed in the cultivar Bowman background have been instrumental in gene localization and identification [18, 19]. Of those, NILs BW849, BW850, and BW851 carry allelic mutations of *single internode dwarf 1* (*sid1*) [19]. The *sid1* mutants exhibit a unique phenotype in which only the peduncle elongates, while other internodes remain condensed due to clustered nodes. To elucidate the molecular mechanisms underlying stem development, we genetically characterized and mapped the *sid1.b* mutation in this study, laying the groundwork for cloning the responsible gene.

## Methods

### Plant materials

A segregating F_2_ population of 1178 plants was derived from a cross between Bowman (wild type) and BW850 (the *sid1.b* mutant) for genetic mapping of the *Sid1* gene. Plants were grown in a greenhouse at 25 °C under 16 h light/8 h dark conditions. Phenotyping was conducted at the boot stage. Critical F_2_ recombinant plants delimiting the *Sid1* region had their phenotype confirmed with at least 30 F_3_ individuals. The *sid1.b* mutation was contributed by GSHO2478, which was a sodium azide induced mutant of Birgitta. The *Sid1* candidate gene was also tested in allelic mutants and their corresponding donors which included BW849 (*sid1.a*) and Akashinriki, and BW851 (*sid1.c*) and Steptoe. Seeds for all genotypes were obtained from the USDA-ARS National Plant Germplasm System (NPGS).

### DNA extraction

DNA was extracted using the CTAB protocol [20] from 100 mg leaf tissue samples collected from each plant. DNA was quantified using a NanoDrop spectrophotometer (NanoDrop 8000, Thermo Fisher Scientific). Final concentration was adjusted to 50 ng/uL for PCR.

### SNP genotyping and marker development

A barley 50 k iSelect SNP Array [21] was used for initial genotyping of forty-eight F_2_s (26 mutants and 22 WTs) and parental lines. Genotype calling was performed with the de novo calling algorithm in GenomeStudio (Illumina, Inc) and marker positions based on pseudo-molecule assembly of Morex V3 [22]. SNPs linked to the phenotype were converted to semi-thermal asymmetric reverse PCR (STARP) markers to genotype the remaining F_2_ population [23]. Polymorphisms were visualized on a 6% polyacrylamide gel stained with GelRed™ nucleic acid stain (Millipore Sigma) and imaged using a Typhoon™ FLA 9500 variable mode laser scanner (GE Healthcare Life Sciences, Marlborough, MA). Markers used are listed in Table 1.

**Table 1:** Marker primers used for genetic mapping of *Sid1*

Marker name	SNP or polymorphism source	Marker type	Forward primer 1	Forward primer 2	Reverse primer
M1	JHI-Hv50k-2016–258154	STARP	GCAACAGGAACCAGCTATGACCCTACCACCACCACCGATCTCAC	GACGCAAGTGAGCAGTATGACCCTACCACCACCACCGATCCAAA	TCGATGATGGGTTCTTGGCT
M2	i_11_10309	STARP	GACGCAAGTGAGCAGTATGACAGAGGAAACCAAAGACCAAA	GCAACAGGAACCAGCTATGACAGAGGAAACCAAAGACACAG	CGGAGGCCGACCGTTAC
M3	JHI-Hv50k-2016–258979	STARP	GACGCAAGTGAGCAGTATGACAAGAGACCTGTTTGTGACCT	GCAACAGGAACCAGCTATGACAAGAGACCTGTTTGTGCACC	TTGGACCTTGCTTCAAAGAATGAC
M4	JHI-Hv50k-2016–259362	STARP	GACGCAAGTGAGCAGTATGACTGTGGTTGCAATTGTATCTGTCTA	GCAACAGGAACCAGCTATGACTGTGGTTGCAATTGTATCTGTACG	TGGCAACAATTCCCTCTGGT
M5	JHI-Hv50k-2016–259377	STARP	GACGCAAGTGAGCAGTATGACAAGATAAGAGCACCGCGTT	GCAACAGGAACCAGCTATGACAAGATAAGAGCACCGAGCC	TGCACATGTTGGAGCAAGCA
M6	JHI-Hv50k-2016–259764	STARP	GCAACAGGAACCAGCTATGACAGCATCCAAGGATGCACTATCG	GACGCAAGTGAGCAGTATGACAGCATCCAAGGATGCACTACAA	ATCGCTGAACTTGCTGGTAC
M7	JHI-Hv50k-2016–261211	STARP	GACGCAAGTGAGCAGTATGACGGTTCCTAGAGGTGGTTCCTT	GCAACAGGAACCAGCTATGACGGTTCCTAGAGGTGGTTCTCC	ACGTCACCTGAGGCTTTGA
M8	JHI-Hv50k-2016–259052	STARP	GACGCAAGTGAGCAGTATGACTTAGTGTAAACAAAAATGATTTCTCT	GCAACAGGAACCAGCTATGACTTAGTGTAAACAAAAATGATTTCCTC	ACCTCCACATATACGGGTATGTG
M9		SSR	AAGACTCGACATGATTCAAAA		TCTTGTAGTATCCTGCCACAC
M10		SSR	ATATCTACGTTTTTGCCCAGT		TGATCTCAATCATGCTAGCTC
M11		SSR	AGCTAGCTACATGCTACAACA		TGTTGGAATTCTTTTTACAGG
M12	JHI-Hv50k-2016–259398	STARP	GACGCAAGTGAGCAGTATGACAATTCGTACCCAATTACCACAT	GCAACAGGAACCAGCTATGACAATTCGTACCCAATTACCAACC	TGCATCTGATTCTGGAATGGC
M13	JHI-Hv50k-2016–259408	STARP	GACGCAAGTGAGCAGTATGACTTATATTTAGAAACGGAGGGAGCAA	GCAACAGGAACCAGCTATGACTTATATTTAGAAACGGAGGGAGTCG	AAGTTGAAGGGTGAATGCTCAT

### Physical mapping and gene prediction

The whole genome sequence assembly of barley cv Morex v3 was used for marker localization and physical mapping [22]. The web-based programs FGENESH [24] and Pfam 32.0 [25] were used for gene prediction and annotation respectively. Gene annotation was confirmed with the BLASTP program and cross-validated with the Morex V3 reference [22]. The gene structure was also verified using the BaRTv1.0 transcript dataset [26].

### SNP calling based on Illumina Genome sequencing

Whole-genome sequencing libraries for Birgitta and BW850 were prepared using the transposase-mediated Nextera Sequencing Library Kit (Illumina, Inc.) and subjected to paired-end sequencing on a HiSeq 4000. Reads were quality-filtered and trimmed using BBDuk (BBTools, https://jgi.doe.gov/data-and-tools/bbtools) [27], then aligned to the Morex reference genome assembly v3 with Bowtie 2 [28]. Duplicate alignments were marked with Samblaster [29]. Reads aligning to the candidate *Sid1* region with a mapping quality (mapQ) score of at least 40 (near-unique) were processed using SAMtools/BCFtools to identify population-level single nucleotide polymorphisms (SNPs) and small insertions/deletions [30]. The resulting VCF file was annotated with SnpEff to assess the predicted effects of polymorphisms on genes within this region [31].

## Results

### Phenotype of the *sid1* mutant

**Fig. 1:**
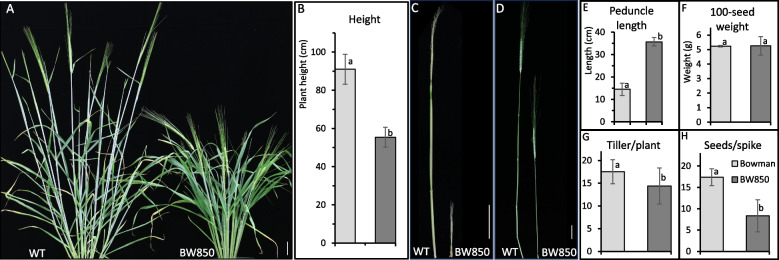
Phenotypic comparison between Bowman (WT) and BW850 (*sid1*). The *sid1* mutant exhibits a stunted phenotype due to impaired stem elongation (**A**). Plant height is significantly reduced in *sid1* compared to WT (**B**). In BW850, stem elongation remains arrested until booting, whereas the third internode is already visible in WT at the same stage (**C**). At the seed-filling stage, the peduncle is the only internode that elongates in BW850 (**D**) and is significantly longer than that in WT (**E**). The difference in 100-seed weight between Bowman and BW850 is not statistically significant (measured with five replications, **F**. However, both tiller number (measured with 10 plants, **G** and seeds per spike (measured with 20 spikes, **H** are significantly reduced in *sid1* compared to WT. Different letters indicate statistically significant differences by T-test (*P* < 0.05). Scale bars, 5 cm

During stem extension, the internodes of BW850 plants carrying the *sid1.b* mutation exhibit defective elongation (Figs. 1a-c). However, the peduncle of BW850 starts elongating at the booting stage and eventually forms a long internode which is approximately 2.5 times longer than that of Bowman (Fig. 1d), while the lower internodes remain arrested (Figs. 1a-c). As a result, the mutant plant at the seed-filling stage is only about half the height of WT (Fig. 1e). Despite similar 100-seed weight between BW850 and Bowman (Fig. 1f and additional Fig S1a), both tiller/plant (Fig. 1g) and seed/spike (Fig. 1h) are significantly lower in BW850. Strikingly, the seed number/spike in the mutant is reduced to nearly one-third of that in WT, with the majority of spikelet being sterile in BW850 (Fig. 1h and additional Fig S1b). Therefore, the *sid1* mutation systemically affects both vegetative and reproductive growth in barley.

### Genetic and physical mapping of the *Sid1* gene

A total of 1034 F_2_ plants derived from the cross between Bowman and BW850 were used for genetic mapping of *Sid1*. Of those, 265 showed the mutant phenotype characterized by a single elongated internode, the peduncle. The observed segregation ratio of mutant to WT fits a 1:3 ratio (χ^2^ = 0.218, df = 1, and *P* = 0.64), suggesting that the *sid1* mutation is monofactorial recessive. To localize the *Sid1* gene and simultaneously identify linked markers, we performed SNP array analysis on 48 F_2_ plants (26 mutants and 22 WTs) along with the parental lines (Additional Table 1). As the *sid1* mutation was previously anchored to 4H [19], we focused on the SNPs on 4H and identified 173 closely linked polymorphic markers to *Sid1* on this chromosome (Additional Table 1).

**Fig. 2:**
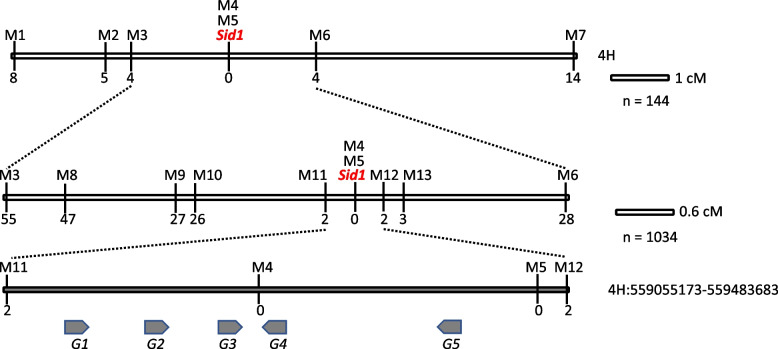
Genetic and physical mapping of the *Sid1* gene. Genetic mapping was conducted sequentially with 144 (**A**) and 890 (**B**) F_2_ individuals. *Sid1* is located on 4H, delimited to an ~ 0.7 cM region between markers M11 and M12 (**B**). A total of six protein-coding genes were identified in the *Sid1* region, spanning ~ 429-kb (**C**). Numbers above the linkage group indicate the number of recombination breakpoints separating the marker from *Sid1*. The maps are drawn to scale. M, marker; G, gene

SNP genotyping with 48 F_2_ progenies revealed that the *Sid1* gene was located in an ~ 21.5-Mb region flanked by two SNPs, JHI-Hv50k-2016–258057 and JHI-Hv50k-2016–261703 (Additional Table 1). To increase the mapping resolution, we converted the linked SNPs to STARP markers and designed SSR markers based on the Morex V3 genome sequence. Initial mapping using 144 F_2_ progenies with these markers further delimited the *Sid1* gene to ~ 8.1-Mb by M3 and M6 (Fig. 2a). Expanding the segregating population to 1,034 F₂ plants further refined the *Sid1* locus to a 429-kb region, defined by markers M11 and M12 (Fig. 2b), within which two co-segregating SNPs (M4 and M5) were identified.

Gene annotation and prediction within this fine-mapped interval identified only five putative protein-coding genes based on the reference genome assembly [32], including genes coding for a translation initiation factor (*G1* or *HORVU.MOREX.r3.4HG0402100*), a 60S ribosomal protein (*G2* or *HORVU.MOREX.r3.4HG0402110*), a neurogenic locus notch protein (*G3* or *HORVU.MOREX.r3.4HG0402120*), an epidermal patterning factor-like protein (*G4* or *HORVU.MOREX.r3.4HG0402130*), and a pectinesterase (*G5* or *HORVU.MOREX.r3.4HG0402150*) (Fig. 2c; Table 2). However, no prior reports suggest that members of these gene families are directly involved in stem elongation, making it challenging to determine which of these genes is the strongest candidate for *Sid1*.

**Table 2:** Predicted genes in the *Sid* region

Gene number	Gene model	Homology
*G1*	*HORVU.MOREX.r3.4HG0402100*	Translation initiation factor IF-2
*G2*	*HORVU.MOREX.r3.4HG0402110*	60S ribosomal protein L38
*G3*	*HORVU.MOREX.r3.4HG0402120*	Neurogenic locus notch homolog protein 1
*G4*	*HORVU.MOREX.r3.4HG0402130*	Epidermal patterning factor-like protein
*G5*	*HORVU.MOREX.r3.4HG0402150*	Pectinesterase

### Selection of the candidate gene for *Sid1*

**Fig. 3:**
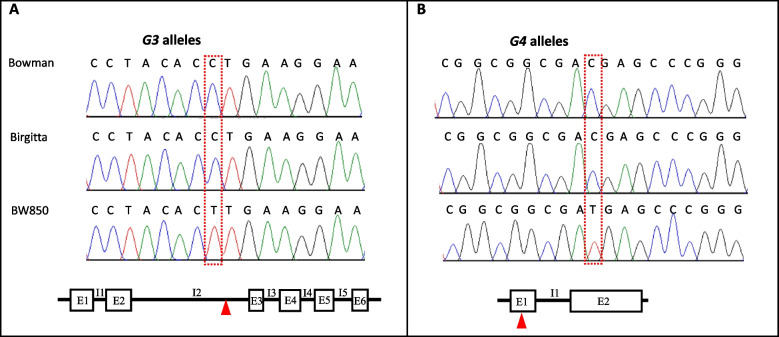
Confirmation of identified SNPs in candidate genes. The C → T transition in *G3* occurs in intron 2 (**A**), but the transition in the first exon of *G4* introduces an early stop codon (**B**)

The original *sid1.b* mutation in GSHO2478 was developed by mutagenesis of barley cv Birgitta using sodium azide, a potent mutagen usually generating point mutations in barley genome [33]. To identify the candidate gene for *Sid1*, we performed shotgun whole-genome sequencing of both BW850 and Birgitta. The 585 M reads of BW850 and 510 M reads of Birgitta represent approximately 34 × and 30 × genome coverage, respectively. Within the *Sid1* region flanked by markers M11 and M12, a total of 47 SNPs were identified, with the majority (45 SNPs) located in intergenic regions (Additional Table 2). There was one each C → T transition in the predictive intron 2 (I2) of *G3* and exon 1 (E1) of *G4* (Table S2), both of which were confirmed by Sanger sequencing (Fig. 3).

Sequencing of *G3* cDNA verified that the intron containing the C → T transition was spliced out of the full-length mRNA, indicating that the mutation in *G3* may not affect plant phenotype (Fig. 3A). In contrast, the C → T transition in *G4* introduced a premature stop codon adjacent to the start codon, leading to complete loss of gene function in the BW850 mutant (Fig. 3B). To further support *G4* as a strong candidate, we used PCR analysis to test allelic mutations of *sid1*, including *sid1.a* in BW849 and *sid1.c* in BW851 [19]. The *sid1.a* mutation was caused by X-ray irradiation using barley cv. Akashinriki; whereas *sid1.c* was generated by fast neutron mutagenesis using Steptoe [19]. In comparison with their respective mutation donors, the *G4* alleles could not be amplified in BW849 and BW851 using various primer combinations targeting regions within, upstream, or downstream of the coding sequence, suggesting this gene was missing in both mutants (Additional Fig. 2). Therefore, *G4* (*HORVU.MOREX.r3.4HG0402130*) encoding an epidermal patterning factor like (EPFL)-protein was selected as a strong candidate for the *Sid1* gene. Sequence alignment revealed that the putative coding product of *G4* is closely related to Arabidopsis EPFL4/5/6 (At4G14723/At3 g22820/At2 g30370) and OsEPFL6 (LOC_Os03 g06610). Accordingly, the candidate gene *G4* was referred to as *HvEPFL6*.

**Fig. 4:**
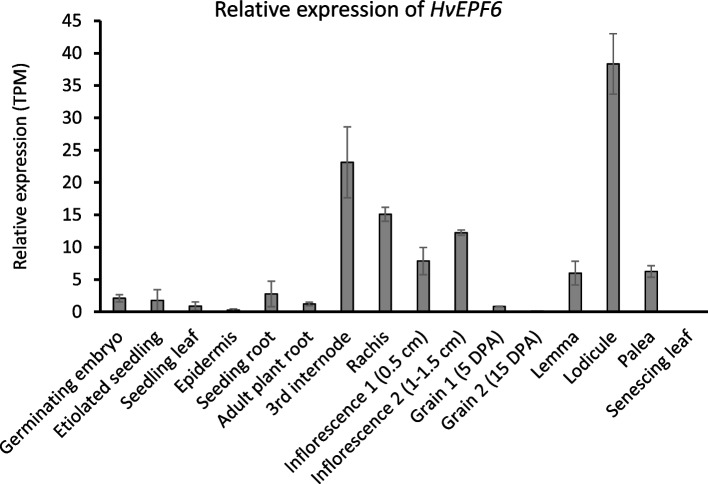
Spatiotemporal quantification of *HvEPFL6* expression based on the barley reference transcript dataset, BaRTv1.0. The plot represents transcript abundances as transcripts per million (TPM) across 16 samples

The BaRTv1.0 transcript dataset provides a high-quality and non-redundant reference database for spatiotemporal quantification of barley genes [26]. The RNA-seq data of BaRTv1.0 showed relatively higher expression levels of *G4* in inflorescence tissues. Among vegetative organs, *G4* expression is specifically enriched in internodes (Fig. 4). Therefore, the expression profile of *G4* indicated its involvement in internode elongation and reproductive growth, which is consistent with the phenotype of *sid1* mutants.

## Discussion

Stem elongation is a key determinant of plant height, which is critical for lodging resistance and the development of an optimal crop ideotype. To elucidate the molecular mechanism underlying stem elongation, we characterized the *sid1* mutant in barley and localized the causal gene for functional validation. Distinct from the *zeo1.b* semidwarf mutant in barley, which is characterized by short internodes (17), loss of *Sid1* function specifically suppressed elongation of the internodes below the peduncle. Yield components are also negatively affected in *sid1*. Genetic mapping narrowed down the *Sid1* locus to a 429-kb region containing five non-transposon protein coding genes, none of which are reportedly associated with stem elongation. Sequencing analysis using Illumina and Sanger methods revealed a C → T transition in *G4* (*HORVU.MOREX.r3.4HG0402130*), introducing a premature stop codon that disrupted gene function in the mutant. This gene encodes an EPF-like protein, designated *HvEPFL6*, which was selected as the *Sid1* candidate gene. Further, deletion of *HvEPFL6* in allelic *sid1* mutants reinforced its candidacy as the causal gene.

EPFs are small secretory cysteine-rich peptides (CRPs) sized less than 160 aa which contain a conserved N-terminal signal sequence and 4–16 cysteine residuals at C-terminal [34]. These small CRPs have been implicated in various physiological processes, such as cell communication, root development, abiotic stresses, plant growth, organ morphogenesis, and fertilization [35, 36]. In *Arabidopsis thaliana*, a total of 11 EPF members have been identified, with EPF1, EPF2, and Stomogen (also known as EPFL9) playing important roles in stomatal development [37–40]. These EPFs act antagonistically with EFP1 and 2 suppressing but Stomogen/EFPL9 promoting stomatal development through functioning as ligands to competitively bind to the ERECTA (ER)-family receptor kinases [41]. However, stomatal development and density in *sid1* seems to be unaffected by the loss of HvERFL6 (Additional Fig. 3). Additionally, EPF members are involved in shaping inflorescence development. With functional redundance, where EPFL4 and EPFL6 function redundantly by physically interacting with ER receptors to regulate inflorescence architecture in *Arabidopsis* [42].

The role of EPFs in plant growth and development has also been demonstrated in cereals. A total of 11 EPF/EPFL members were identified in both rice and barley [43, 44], 12 in rye (*Secale cereale* L.) [45], and 15 in maize (*Zea mays* L.) [46]. Overexpression of *ERF1* indicated that EPF1-supprssed stomatal development is conserved in rice and barley, enhancing drought tolerance without yield penalties [47, 48]. In addition, the loss of OsEPF1 function during domestication led to an increased number of grains per panicle, shorter grains, and an awnless phenotype in cultivated rice [49]. CRISPR-mediated mutagenesis further revealed that OsEPFL2, OsEPFL7, OsEPFL9, and OsEPFL10 regulate awn length in rice [43]. Acting as ligands for OsER1, OsEPFL6, OsEPFL7, OsEPFL8, and OsEPFL9 function synergistically to negatively regulate grain number per panicle [50]. These findings highlight the evolutionary conservation of EPF–ER ligand-receptor interactions in plant development, particularly reproductive growth, across both dicot and monocot species.

Genetic and physical mapping in the present study suggested the *sid1* phenotype in barley might result from the loss of *HvEPF6* function. While EPF-mediated development is well-documented, the arrested internode elongation below the peduncle caused by EPF mutation appears to be unique to barley. It was suggested that a neofunctionalization of EPF members may have occurred during barley speciation. However, confirmation of *Sid1* as *HvEPFL6* will require genetic transformation or CRISPR/Cas9-mediated mutagenesis in barley, and *Sid1* cloning will facilitate the identification of its receptor. Therefore, fine mapping of *Sid1* in this study provides a candidate for functional validation, laying the foundation to advance our understanding of the molecular mechanism underlying stem elongation in monocots.

## Conclusions

In summary, we have characterized and genetically mapped the *sid1.b* mutation causing a dwarfed phenotype single internode in barley. Our results indicate that the *sid1* mutation imposes a systemic effect on barley growth and development. The *Sid1* gene was mapped to chromosome 4H within a 429-kb region. Illumina sequencing of WT and *sid1* identified a C → T transition in the *HvEPFL6* gene, which introduces a premature stop codon in the mutant allele. Therefore, our study provides a foundation for cloning of *Sid1*, which will enhance our understanding of the molecular mechanisms underlying stem development, particularly in monocot plants.

## Supplementary Information

Supplementary Material 1.

Supplementary Material 2.

Supplementary Material 3.

## Data Availability

The re-sequencing data in this manuscript have been deposited in NCBI Sequence Read Archive under accession number PRJNA1247420. All data generated or analyzed during this study are included in this published article and its supplementary information files.
