# Supplementary material for: Genetic analysis of the single internode dwarf 1 mutant in barley
Source: BMC Plant Biol. 2025 Jul 2;25:797. doi: 10.1186/s12870-025-06790-6 (PMC12220251; doi:10.1186/s12870-025-06790-6)
Supplement: Supplementary file 3 — Supplementary Material 3. [file 12870_2025_6790_MOESM3_ESM.pptx]

## Slide 1
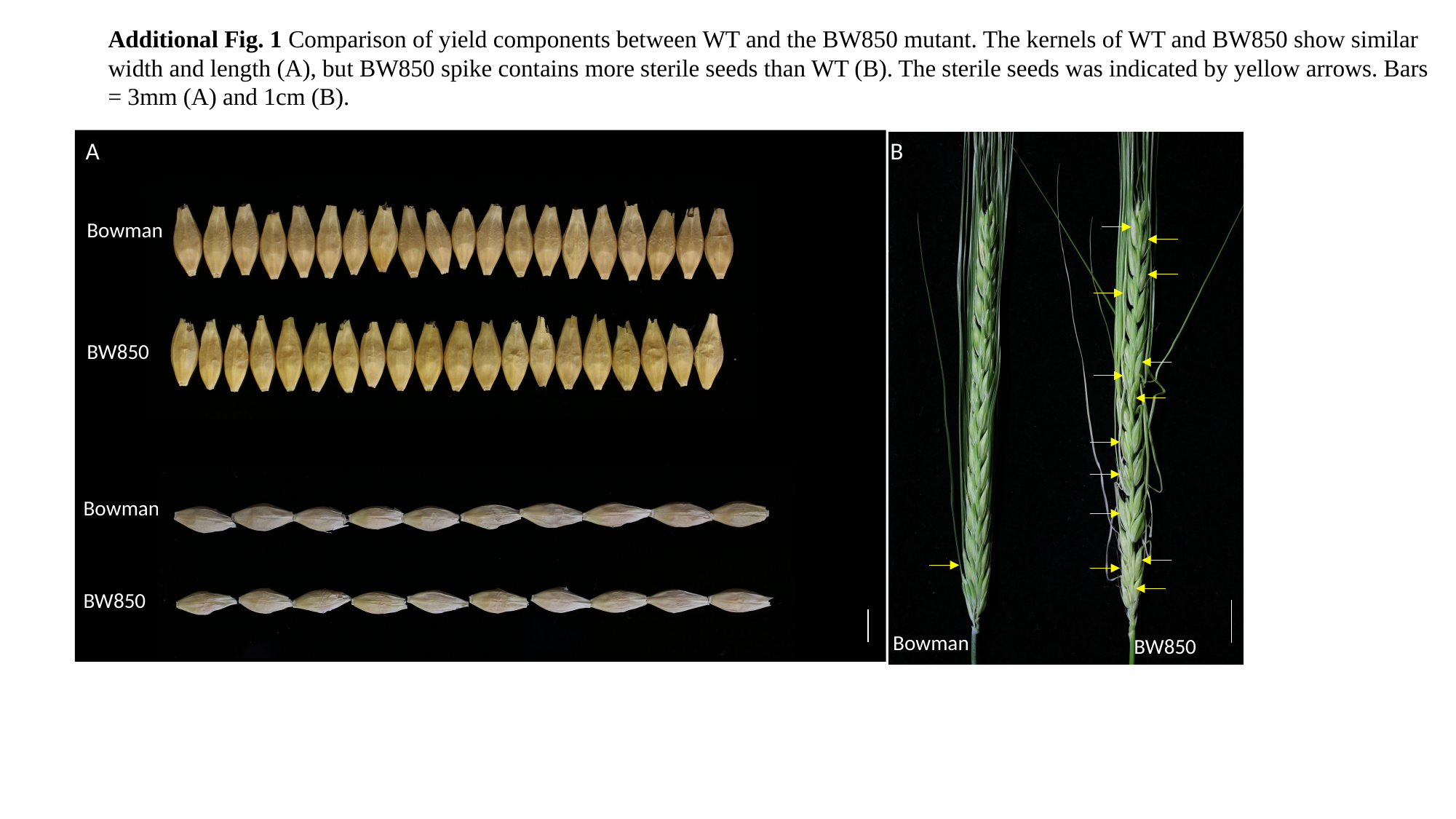

Additional Fig. 1 Comparison of yield components between WT and the BW850 mutant. The kernels of WT and BW850 show similar width and length (A), but BW850 spike contains more sterile seeds than WT (B). The sterile seeds was indicated by yellow arrows. Bars = 3mm (A) and 1cm (B).
A B
Bowman
BW850
Bowman
BW850
Bowman
BW850

## Slide 2
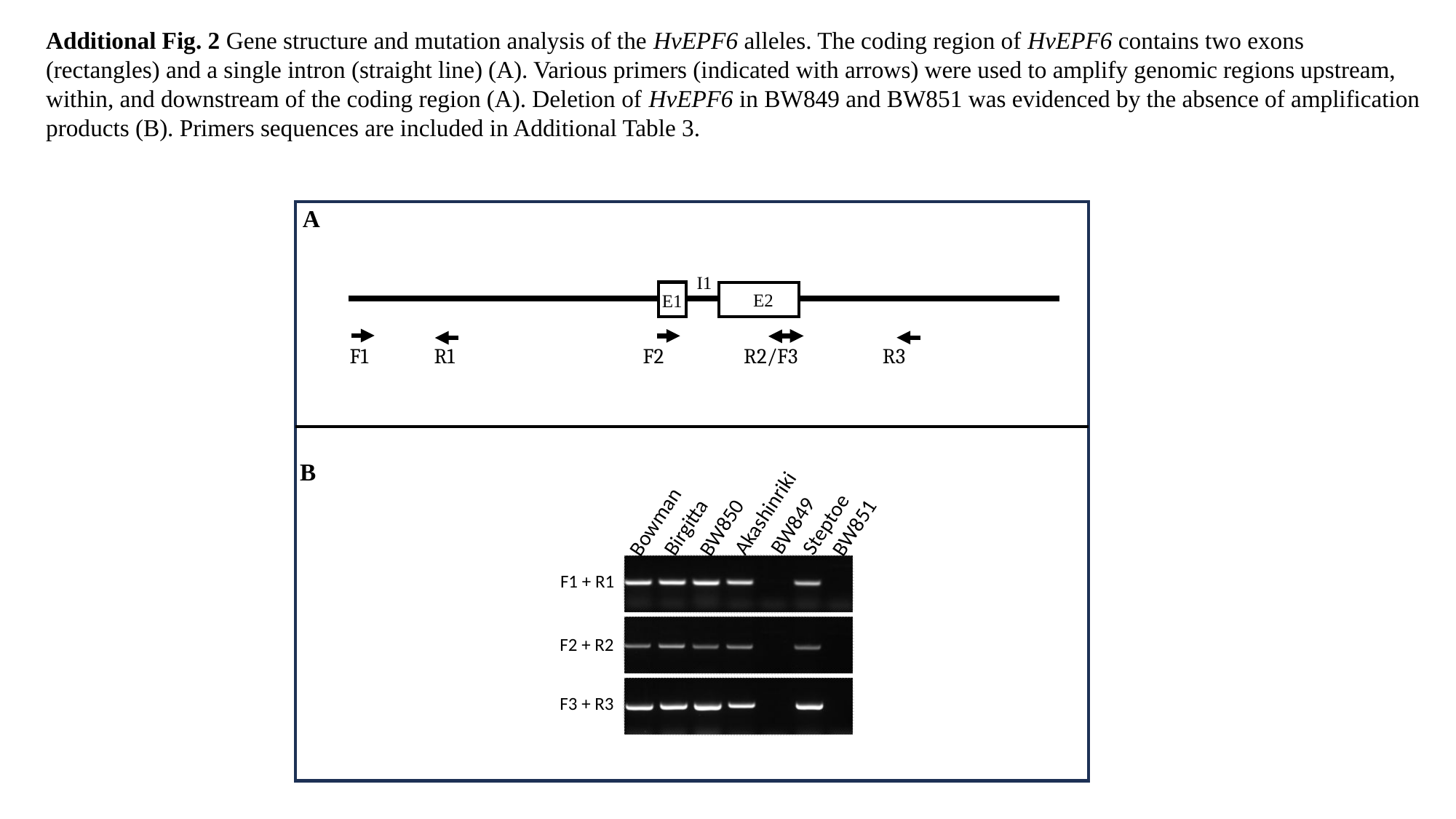

Additional Fig. 2 Gene structure and mutation analysis of the HvEPF6 alleles. The coding region of HvEPF6 contains two exons (rectangles) and a single intron (straight line) (A). Various primers (indicated with arrows) were used to amplify genomic regions upstream, within, and downstream of the coding region (A). Deletion of HvEPF6 in BW849 and BW851 was evidenced by the absence of amplification products (B). Primers sequences are included in Additional Table 3.
A
I1
E2
E1
F1 R1 F2 R2/F3 R3
BW849
Steptoe
Akashinriki
Birgitta
BW850
Bowman
BW851
F1 + R1
F2 + R2
F3 + R3
B

## Slide 3
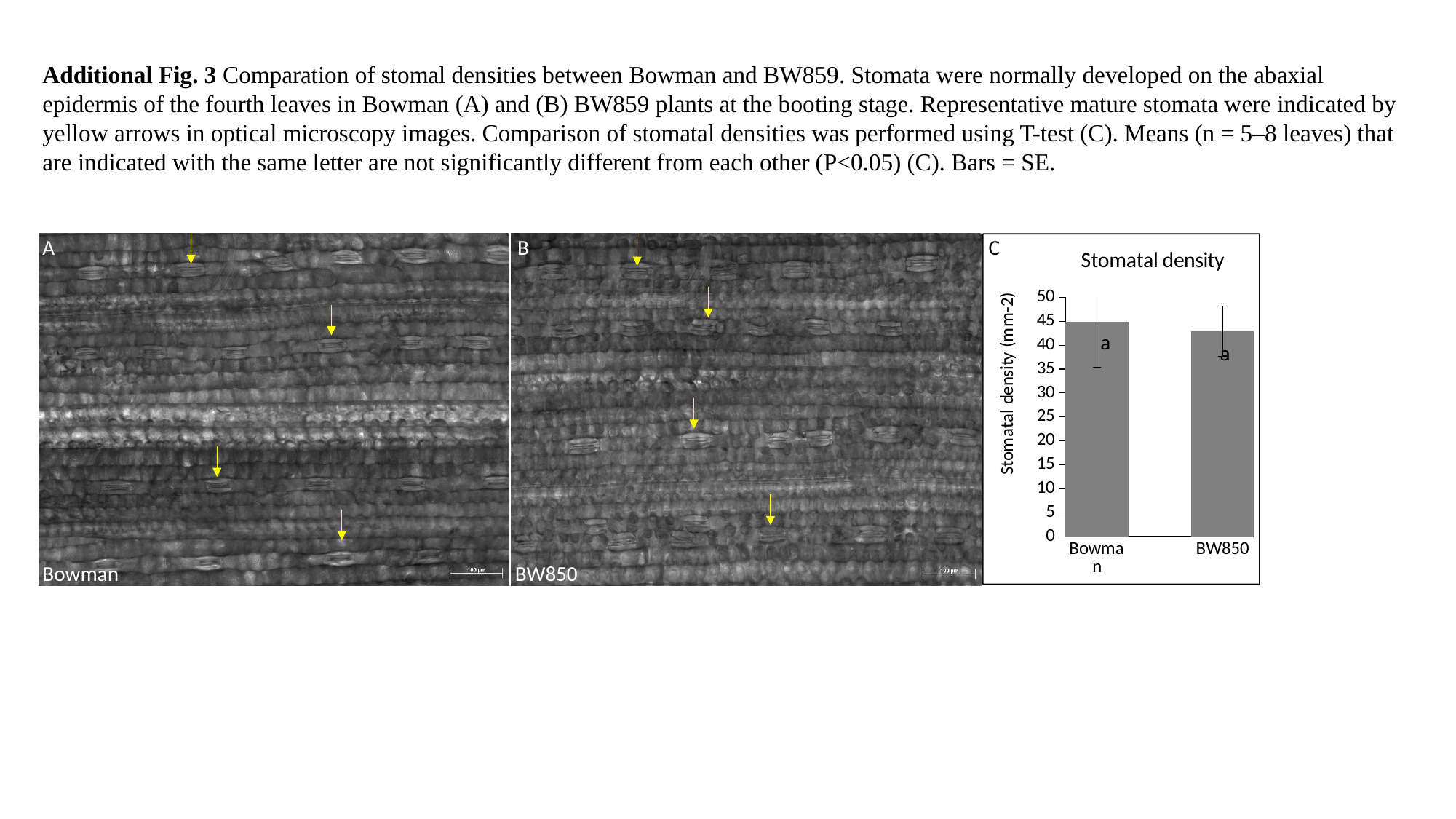

Additional Fig. 3 Comparation of stomal densities between Bowman and BW859. Stomata were normally developed on the abaxial epidermis of the fourth leaves in Bowman (A) and (B) BW859 plants at the booting stage. Representative mature stomata were indicated by yellow arrows in optical microscopy images. Comparison of stomatal densities was performed using T-test (C). Means (n = 5–8 leaves) that are indicated with the same letter are not significantly different from each other (P<0.05) (C). Bars = SE.
A B C
### Chart: Stomatal density
| Category | |
|---|---|
| Bowman | 44.9024086378738 |
| | None |
| BW850 | 42.9233284883721 |a
a
Bowman BW850

## Slide 4
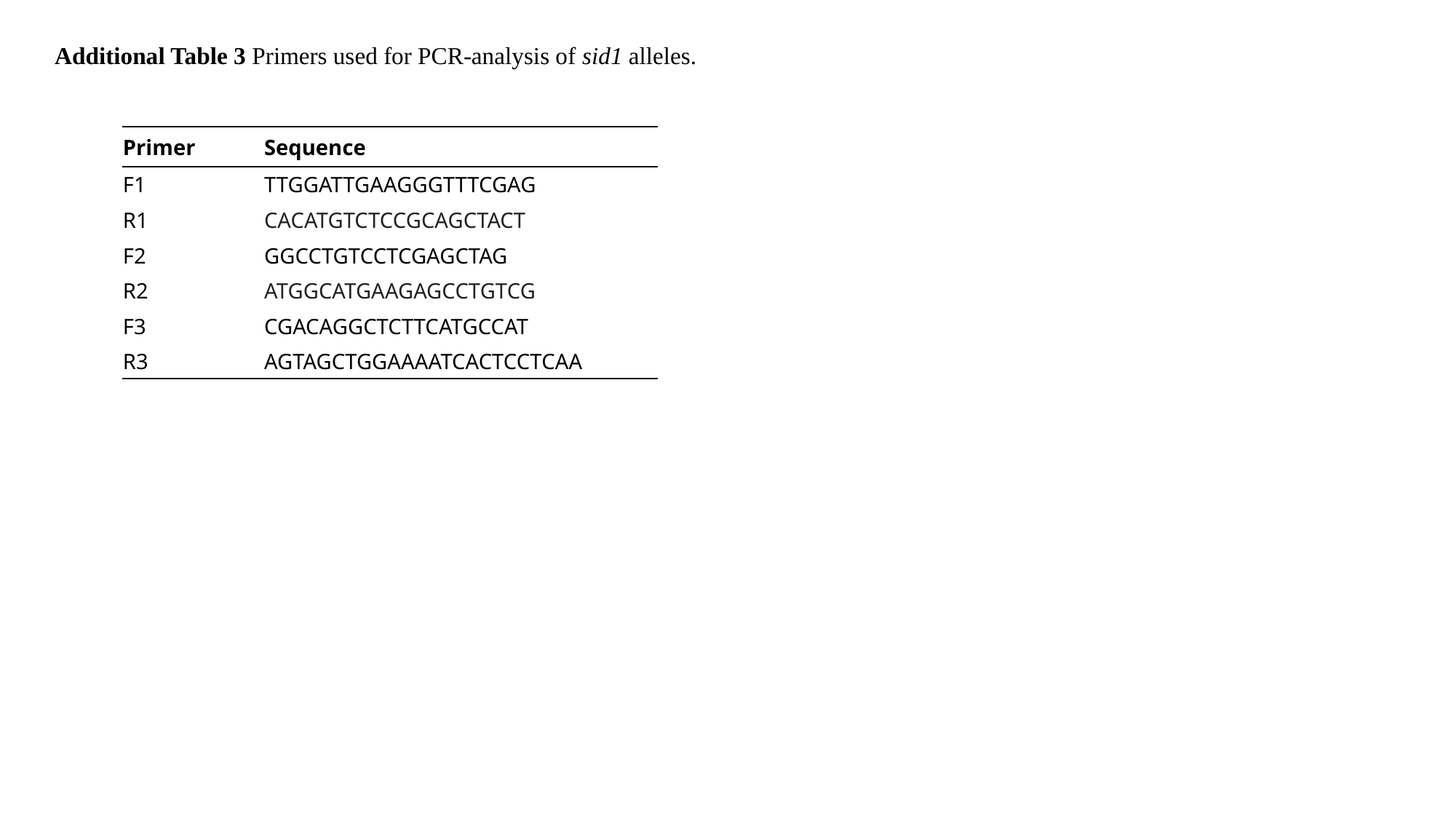

Additional Table 3 Primers used for PCR-analysis of sid1 alleles.
| Primer | Sequence |
| --- | --- |
| F1 | TTGGATTGAAGGGTTTCGAG |
| R1 | CACATGTCTCCGCAGCTACT |
| F2 | GGCCTGTCCTCGAGCTAG |
| R2 | ATGGCATGAAGAGCCTGTCG |
| F3 | CGACAGGCTCTTCATGCCAT |
| R3 | AGTAGCTGGAAAATCACTCCTCAA |
